# Modified wideband 3D late gadolinium enhancement (LGE) MRI for patients with implantable cardiac devices

**DOI:** 10.1186/1532-429X-17-S1-Q26

**Published:** 2015-02-03

**Authors:** Shams Rashid, Stanislas Rapacchi, Kalyanam Shivkumar, Adam Plotnik, Paul J Finn, Peng Hu

**Affiliations:** 1 Radiological Sciences, University of California, Los Angeles, Los Angeles, CA, USA; 2 UCLA Cardiac Arrhythmia Center, University of California, Los Angeles, Los Angeles, CA, USA; 3 Biomedical Engineering Interdepartmental Program, University of California, Los Angeles, Los Angeles, CA, USA

## Background

Late gadolinium enhancement (LGE) cardiac MRI is the clinical gold standard for non-invasive assessment of myocardial viability and plays an important role in guiding catheter ablation of ventricular tachycardia (VT)^1^. The majority of VT patients have implanted cardiac devices such as implantable cardioverter defibrillators (ICDs). The presence of ICDs gives rise to strong off-resonance within the myocardium. This produces hyper-intensity (HI) artifacts in LGE, which can mask scar tissue, compromising the diagnostic value of LGE^2^. Recent studies show that HI artifacts can be eliminated by using a wideband inversion recovery (IR) pulse in the LGE sequence^2-3^. However, the current wideband LGE is a 2D sequence, which limits spatial resolution, especially slice thickness (8 mm). This is problematic for using LGE to guide catheter ablation of VT. High resolution LGE is feasible using a 3D LGE sequence. However, no prior studies have explored 3D LGE under the influence of strong off-resonance imposed by ICDs.

## Methods

We implemented the wideband IR pulse (bandwidth (BW) = 3.8 kHz) in a 3D LGE sequence (old wideband 3D LGE). Initial testing revealed extended signal void and banding artifacts, which were not present in corresponding 2D wideband LGE images. We found that these artifacts are caused by slab distortion due to off-resonance produced by the ICD. Slab distortion is inversely proportional to slab select gradient (Gss), so increasing Gss will reduce artifacts. To increase Gss, RF excitation pulse BW also needs to be increased. We modified the sequence by increasing the RF BW from 5.8 kHz to 12 kHz (modified wideband 3D LGE). We evaluated the new sequence in 6 volunteers (an ICD was attached to the left shoulder) and in 5 ICD patients. Image scoring (1-4 scale, 1=best) was used to compare artifacts between the old and modified wideband 3D LGE images in volunteers, and image quality between wideband 2D and modified wideband 3D LGE images in the ICD patients. Scores were compared using a Wilcoxon signed-rank test.

## Results

**Figure 1:**
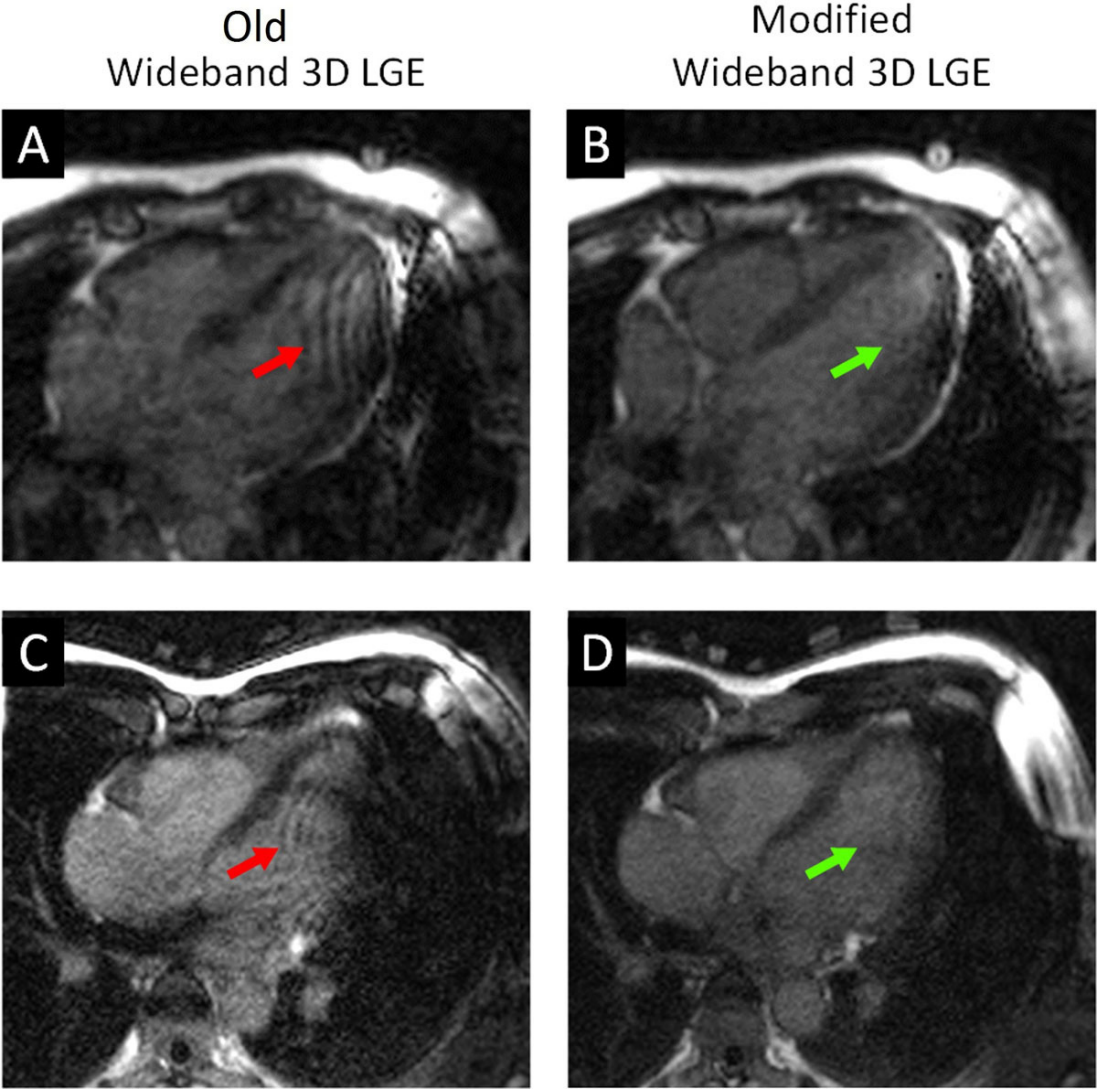
Ripple artifacts (A, *red arrow*) and extended signal voids (C, *red arrow*) that were formed in the myocardium in two healthy volunteers using the old wideband 3D LGE sequence. Both artifacts were substantially reduced using the modified wideband 3D LGE sequence (B & D, *green arrows*). An ICD was attached to the left shoulder of the volunteers.

**Figure 2:**
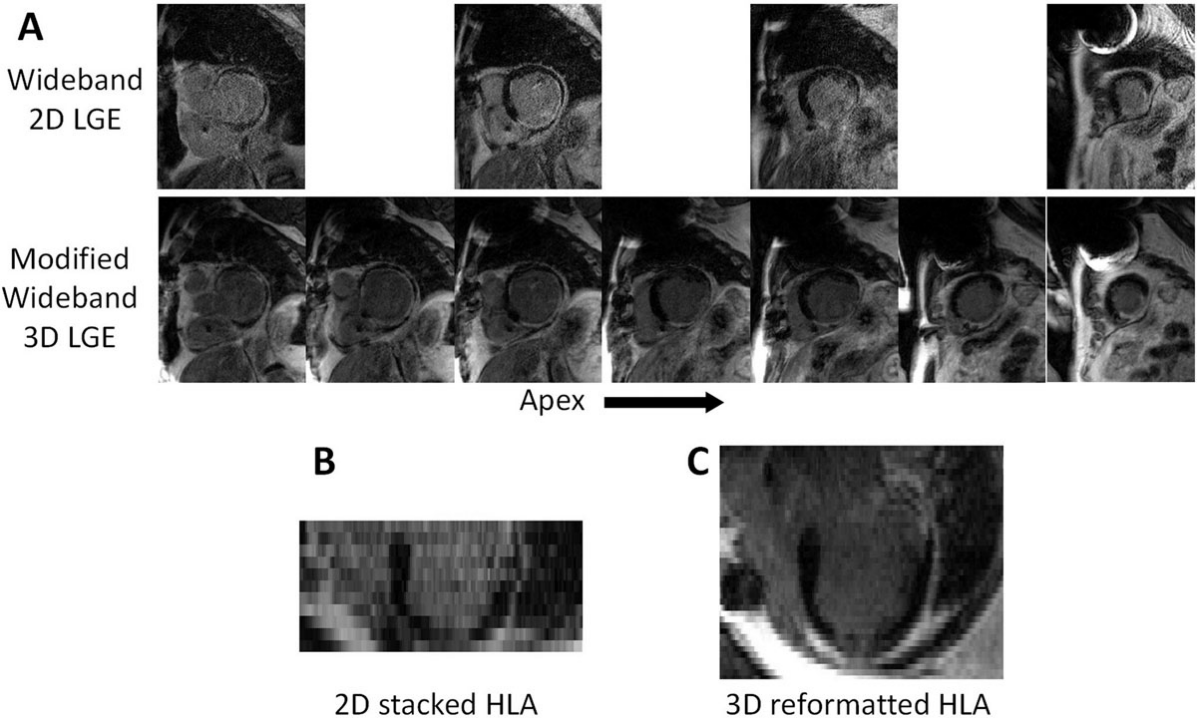
A: Comparison between the clinically used wideband 2D LGE sequence and the modified wideband 3D LGE sequence at the same slice locations of the myocardium in an ICD patient who was scheduled for VT ablation. The 3D images appear less noisy and provide better coverage of the myocardium and scar regions. The ICD is located at the upper-left of the image. B & C: Four-chamber HLA images created from the 2D (B) and 3D (C) LGE images. In (B), the 2D LGE images were stacked to produce the volume, and reformatted to the HLA view. The large slice thickness and slice misregistration is evident in the 2D reformatted image (B), while the 3D volume and higher slice resolution in (C) produces a better HLA image.

Extended signal void and banding artifacts in volunteer images were significantly reduced using the modified wideband 3D LGE sequence (Fig. 1): artifact score improved from 2.7±0.6 in the old wideband 3D LGE to 2.2±0.8 in the modified wideband 3D LGE images (p<0.05). In the ICD patients, the modified wideband 3D LGE produced images that had higher resolution (1.4x1.4x4 mm^3^ vs. 1.9x1.4x8 mm^3^) and similar image quality scores (p=0.46) as wideband 2D LGE (Fig. 2).

## Conclusions

We developed a modified 3D wideband LGE sequence, which reduces off-resonance slab distortion artifacts that occur in conventional 3D LGE. Our results in ICD patients yielded similar image quality and higher spatial resolution compared to wideband 2D LGE. This represents a first step towards high resolution 3D LGE imaging in ICD patients in a clinical setting.

## Funding

NIH 1R21 HL118533.
